# Comprehensive evaluation of TIR 3B thyroid nodules: Clinical, Ultrasonographical and Cytological features for prediction of malignancy

**DOI:** 10.1007/s12020-026-04597-5

**Published:** 2026-04-01

**Authors:** Ignazio Barca, Antonio Prinzi, Guenda Di Benedetto, Francesco Arcidiacono, Tommaso Piticchio, Dario Tumino, Giuseppe Evola, Maria Antonietta Trovato, Agata Bosco, Ilenia Marturano, Gabriella Pellegriti, Pasqualino Malandrino, Marco Russo, Francesco Frasca

**Affiliations:** 1 Endocrinology Unit, Dept. of Clinical and Experimental Medicine, University of Catania, Garibaldi-Nesima Medical Center, Catania, Italy; 2 Department of Precision Medicine in Medical, Surgical and Critical Care (Me.Pre.C.C.), University of Palermo, Palermo, Italy; 3 Department of Medicine and Surgery, University Kore of Enna, Enna, 94100 EN Italy; 4 Department of General and Emergency Surgery, Garibaldi-Nesima Medical Center, Catania, Italy; 5 Surgical Oncology Department, Garibaldi-Nesima Medical Center, Catania, Italy; 6 Pathology Unit, ARNAS Garibaldi Hospital, Catania, Italy; 7 Medical Oncology, Department of Clinical and Experimental Medicine, University of Catania, Catania, Italy

**Keywords:** Thyroid nodules, Indeterminate cytology, TIR 3B, Thyroid cancer, Ultrasonography, Nomogram

## Abstract

**Purpose:**

Thyroid nodules are common clinical findings, with a subset requiring evaluation for malignancy. TIR 3B nodules, classified as indeterminate with high risk, present a diagnostic challenge. This study aims to identify clinical, ultrasonographic, and cytological features predictive of malignancy in TIR 3B thyroid nodules.

**Methods:**

We retrospectively analyzed 175 patients with TIR 3B nodules who underwent surgery, evaluating their clinical, ultrasound, and cytological characteristics.

**Results:**

The malignancy rate was 43.4%, with papillary thyroid carcinoma being the most prevalent. Multivariable analysis identified solid hypoechoic nodules, irregular margins, nuclear grooves, and micro- and macrocalcifications as independent predictors of malignancy. Based on these features, we developed a predictive nomogram model with an area under the curve (AUC) of 0.790, demonstrating good diagnostic performance. The model identified a malignancy risk cut-off score of 8, with high specificity (94%) for distinguishing benign from malignant nodules.

**Conclusions:**

This study highlighted several findings regarding malignancy risk and identified ultrasonographic and cytological features associated with malignant outcomes to help determine which patients should be candidates for surgery. The proposed predictive model could be a valuable clinical tool and may facilitate more personalized treatment approaches.

## Introduction

Thyroid nodule is a distinct lesion within the thyroid gland that can be differentiated from the surrounding tissue using advanced imaging techniques [1]. Thyroid nodules are a common clinical finding, with a high prevalence in general population [2]. In iodine-sufficient populations, thyroid nodules are detected in approximately 5% of individuals through physical examination (neck palpation), with prevalence varying by age and sex. However, clinicians identify a significantly higher proportion of patients with occult thyroid nodules, affecting up to 68% of the general population [3, 4]. Most of thyroid nodules, up to 90%, are benign, including conditions such as thyroid follicular nodular disease, follicular adenomas, and oncocytic adenomas [1, 5, 6]. Therefore, the primary objective of clinical evaluation is to rule out malignancy, which may include papillary, follicular, oncocytic, medullary, and anaplastic thyroid cancers trying to avoid overdiagnosis and overtreatment [7]. Ultrasound (US) is the most sensitive method for detecting thyroid nodules and is the first-line imaging modality for malignancy risk assessment [8, 9]. Certain ultrasound features, including hypoechogenicity, a taller-than-wide shape, irregular margins, microcalcifications, and extrathyroidal extension, are known to be associated with malignancy [10]. Relying on individual ultrasound features as standalone diagnostic criteria can lead to inter- and intra-operator variability. To address these limitations, several ultrasound risk stratification systems have been developed to assess a nodule’s malignancy risk and guide the need for fine-needle aspiration (FNA) [11–14]. Indeed, the most reliable and common diagnostic procedure for thyroid nodule diagnosis is fine-needle aspiration cytology (FNAC) which accurately diagnoses benign and malignant nodules in most cases [15]. However, a nonnegligible number of FNACs, up to 25%, are classified as indeterminate in which a clear diagnosis can be achieved due to histopathological examination following surgery [16–18]. The indeterminate category is typically divided into two subcategories across different classification systems. In the UK Royal College of Pathologists guidelines, these are Thy 3a (with atypia) and Thy 3f (with a follicular pattern) [19]. The Bethesda System for Reporting Thyroid Cytopathology classifies them as AUS (atypia of undetermined significance) and FN (follicular neoplasm) [20]. Similarly, the Italian Consensus for the Classification and Reporting of Thyroid Cytology (ICCRTC) designates them as TIR 3 A and TIR 3B [21]. The ICCRTC reported an expected risk of malignancy < 10% and 15–30% for TIR 3 A and TIR 3B respectively, but this risk of malignancy is mainly obtained based on clinical experience and is only partially based on the evidence of the published data [21]. A recent systematic review and meta-analysis demonstrated that these subcategories showed a higher risk of malignancy than previous reported, 17% for TIR 3 A and 47% for TIR 3B [22]. This meta-analysis supports the actions suggested from the panel of ICCRTC: repeat FNA or clinical follow-up in presence of a TIR 3 A and surgery in case of TIR 3B. Therefore, despite the high cancer prevalence reported in TIR 3B, still half of the patients with a cytological diagnosis of TIR 3B show a benign thyroid lesion, without the need to undergo surgery. A previous study reported that age < 55 years, nodule size < 20 mm and microcalcifications were features independently related to malignancy in TIR 3B nodules [23]. More data is therefore needed to identify factors associated with malignancy in patients with a cytological diagnosis of TIR 3B. The aim of this study is to identify clinical, ultrasonographic, and cytologic factors that can predict malignancy in indeterminate high-risk thyroid nodules classified as TIR 3B.

## Materials and methods

### Study population and data collection

In this retrospective study, we analyzed data from a series of consecutive patients who underwent FNAC at the Thyroid Clinic of the Garibaldi-Nesima Medical Center, a tertiary referral center (Endocrinology Unit, Department of Clinical and Experimental Medicine, Catania, Italy), between May 2014 and December 2023. All patients evaluated for thyroid-related conditions underwent a comprehensive thyroid hormonal assessment, including measurement of thyroid-stimulating hormone (TSH), free triiodothyronine (FT3), free thyroxine (FT4), and calcitonin levels when clinically indicated, along with a thyroid US examination. Patients diagnosed with TIR 3B nodules were referred for surgical intervention. The inclusion criteria were as follows: (1) FNAC results reporting TIR 3B on cytological examination; (2) Serum calcitonin levels within the normal range; (3) Patients underwent thyroid surgery; (4) Availability of data on clinical, ultrasonographic, cytological, and histopathological characteristics. Exclusion criteria included: (1) Diagnostic tests and follow-up performed in a different clinical setting; (2) TSH levels below 0.5 µIU/mL; (3) Autonomous thyroid nodules identified via scintigraphy. Mandatory data collected for the analysis included patient gender, age at diagnosis, serum thyroid peroxidase (TPO) and thyroglobulin (Tg) antibody status, TSH levels, thyroid function status (euthyroid, hyperthyroid, or hypothyroid), and family history of thyroid cancer. The study adhered to the principles of the Declaration of Helsinki and was approved by the Institutional Review Board and Ethical Committee Catania 2 in Sicily, Italy (approval n. 337/CEL). Informed consent was waived due to the retrospective nature of the study.

### Ultrasound examination

All patients underwent thyroid and neck US using a high-frequency linear probe. US examinations were performed by endocrinologists with a minimum of 10 years of experience. The following US features were collected: maximum diameter (millimeters), margins (i.e.; regular or irregular), taller than wide shape (i.e.: present or absent), echogenicity (i.e.; partially cystic nodule, solid isoechoic, solid hypoechoic, or anechoic), and calcifications (i.e.; absent, micro-calcifications, macro-calcifications).

### Cytological and histopathological examination

FNAC of thyroid nodules was conducted in accordance with established international guidelines [24, 25]. Cytological evaluations of FNAC smears were performed by a cytopathologist specializing in thyroid pathology and classified following the ICCRTC. The histopathological examination was performed by two expert pathologists on thyroid pathology. The following cytological features were reported: colloid, macrophages, oncocytic cells, inflammation, fibrosis, chromatin clearing, fusiform nuclei, nuclear grooves, nuclear inclusions, microfollicular and trabecular structures. According to the SIAPEC-AIT 2014 classification, the high-risk indeterminate category (TIR3B) includes lesions showing some nuclear features suggestive of papillary carcinoma, such as nuclear grooves, nuclear inclusions, or overlapping nuclei, that raise suspicion for malignancy but are too mild, focal, or incomplete to warrant a definitive TIR4 (suspicious for malignancy) diagnosis [21, 23]. The histological findings in this study were reported using the revised nomenclature outlined in the WHO 2022 classification of thyroid neoplasms [26]. In 2016, a new category, *Non-Invasive Follicular Thyroid Neoplasm with Papillary-Like Nuclear Features* (NIFTP), was introduced to classify the encapsulated follicular variant of papillary thyroid carcinoma (EFVPTC) [27]. According to the updated WHO classification, this entity is now regarded as having benign behavior [26]. Therefore, in our study, NIFTP and other low-risk neoplasms were considered as separate categories and were not classified as malignant for the calculation of the risk of malignancy. This approach ensures a more objective estimation of ROM and allows comparison with previous studies in which NIFTP was either included in the malignant category or not consistently reported.

### Statistical analysis

Descriptive statistics were computed for continuous variables, with values expressed as means ± standard deviations (SD) or medians with 25th–75th interquartile ranges (IQR) for variables not following a normal distribution. Categorical variables were presented as frequencies and percentages. Group comparisons for continuous variables were conducted using the t-test for normally distributed variables and the Mann-Whitney U test for non-normally distributed variables. The chi-square test was used for categorical variable comparisons. To evaluate the association between malignancy and clinical, ultrasound and cytological factors, logistic regression analysis was used to calculate odds ratios (ORs) with 95% confidence intervals (CIs) through both univariate and multivariable analyses. All variables found to be statistically significant in the univariate analysis were included in the multivariable models, which were refined using a backward stepwise selection approach. A nomogram was developed based on the final prediction model to estimate the risk of malignancy by assigning a weighted score to each of the predictors identified at the multivariable analysis. The total scores were calculated as a summary of each predictor’s scores, identified by drawing lines to the points axis. The risk of malignancy was estimated by referring to the probability line. Finally, receiver operating characteristic (ROC) curve analysis was employed to determine the cut-off point for the total score that best predicted the risk of malignancy. Statistical significance was set at *p* < 0.05. Statistical analyses were conducted using STATA version 18.5 (StataCorp LP, College Station, Texas, USA).

### Results

#### Clinical features of patients with TIR 3B nodules

**Fig. 1:**
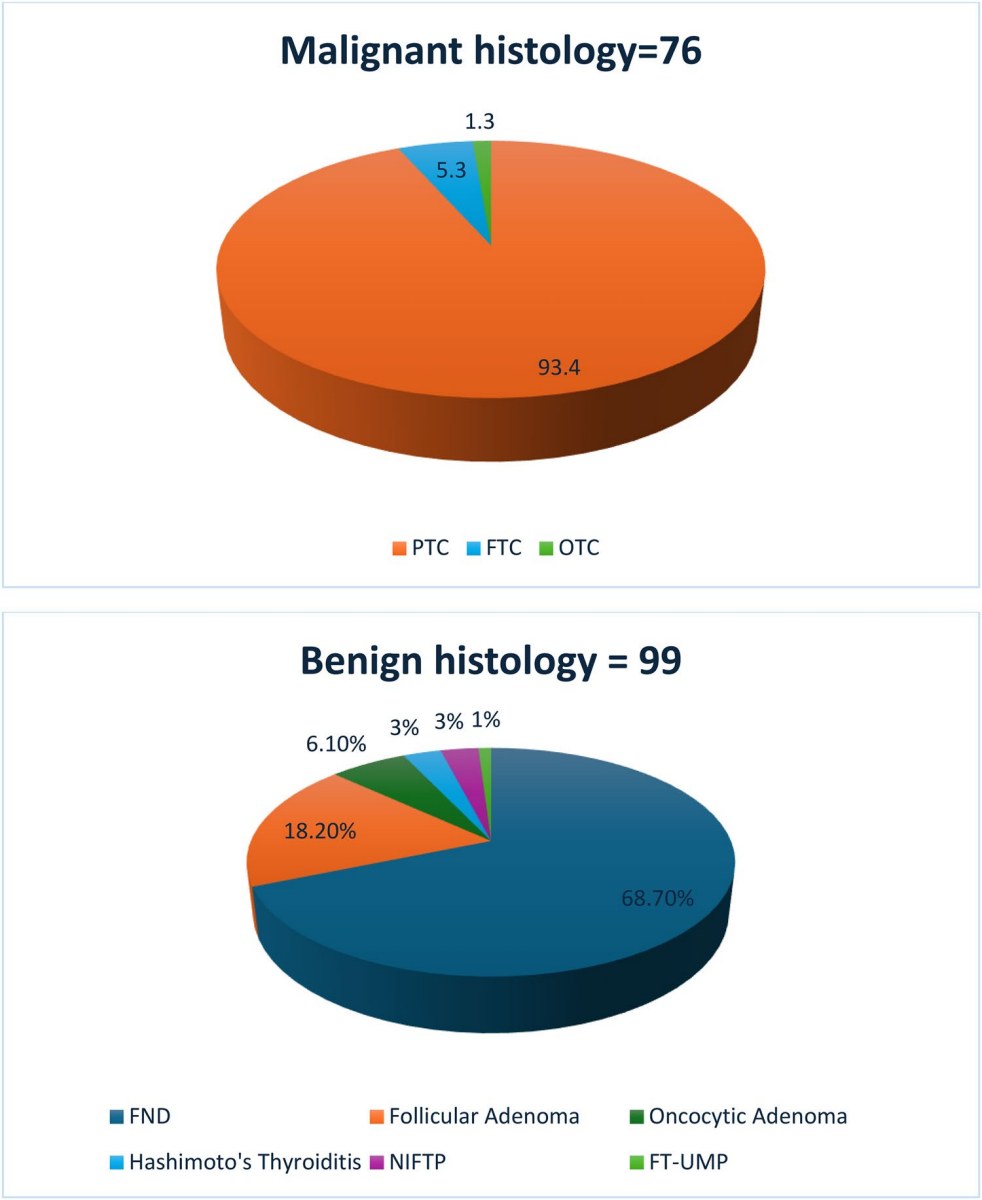
Prevalence of malignant (**A**) and benign (**B**) histological diagnoses of TIR 3B nodules after surgery. PTC, papillary thyroid carcinoma; FTC, follicular thyroid carcinoma; OTC, oncocytic thyroid carcinoma; FND, follicular nodular disease; NIFTP, non-invasive follicular thyroid neoplasms with papillary-like nuclear features; FT-UMP, follicular thyroid tumors of uncertain malignant potential

We collected 586 aspirate samples that were cytologically classified as TIR 3B. A total of 411 participants were excluded due to the absence of histological data or other essential information, as outlined in the exclusion criteria. This left a final cohort of 175 eligible subjects included in the analysis. The mean age at diagnosis was 52.3 ± 13.9 years; 33 patients (22%) were male, and 142 (78%) were female. Among these patients, 161 were euthyroid, 10 (6%) were hypothyroid and receiving levothyroxine replacement therapy, and 4 (2%) had Graves’ disease. The median TSH level was 1.41 (IQR: 0.9–2.21). A family history of thyroid carcinoma was reported in 23 patients (13%). Of the 175 evaluated nodules, 43.4% were confirmed as malignant, with papillary thyroid carcinoma (PTC) being the most prevalent type (93.4%) (Fig. 1). Follicular thyroid carcinoma and oncocytic thyroid carcinoma of the thyroid were less common, representing 5.3% and 1.3% of malignant lesions, respectively. In contrast, 56.6% of the nodules were classified as benign or low-risk neoplasms. The most common benign finding was thyroid follicular nodular disease (FND), accounting for 68.7% of these cases. Follicular adenomas and oncocytic adenomas were present in 18.2% and 6.1% of cases, respectively. Less frequent diagnoses included Hashimoto’s thyroiditis (3.0%), NIFTP (3.0%), and follicular thyroid tumors of uncertain malignant potential (FT-UMP) (1.0%).

#### Comparison between malignant and benign TIR 3B nodules

The clinical, ultrasonographic, and cytological characteristics of 76 patients with malignant TIR 3B nodules were compared with those of 99 patients with benign TIR 3B nodules. Table 1 presents the comparison between the two groups. At diagnosis, no significant differences were observed between the two groups in terms of male-to-female ratio, TSH levels, AbTPO/AbTG positivity, family history of cancer, thyroid function, or the presence of multinodular goiter. However, patients with malignant TIR 3B nodules were significantly younger (49.4 ± 13.2 years vs. 54.5 ± 14.1 years, *p* = 0.016). Among ultrasonographic features, patients with malignant nodules showed a higher incidence of hypoechogenicity (64% vs. 29%, *p* < 0.001), irregular margins (25% vs. 2%, *p* < 0.001), a taller-than-wide shape (9% vs. 2%, *p* = 0.033), macrocalcifications (13% vs. 4%, *p* = 0.028), and microcalcifications (25% vs. 1%, *p* < 0.001), despite no significant difference in nodule size. Malignancy was also strongly associated with chromatin clearing (99% vs. 91%, *p* = 0.028), nuclear grooves (47% vs. 16%, *p* < 0.001), and nuclear inclusions (17% vs. 2%, *p* < 0.001). Conversely, benign nodules exhibited a higher incidence of microfollicular structures (89% vs. 76%, *p* = 0.027).

**Table 1:** Clinicopathological features at diagnosis in patients with benign and malignant TIR 3B nodules and comparison between groups

Parameters	Benign	Malignant	
**Clinical features**	*N* = 99	*N* = 76	P value
Sex, Female, n. (%)	78 (79)	59 (78)	0.85
Age at diagnosis, years (mean ± SD)	54.5 ± 14.1	49.4 ± 13.2	0.016
TSH value, median (25th -75th IQR)	1.3 (0.8–2.2)	1.9 (0.9–2.2)	0.50
AbTPO/AbTG positivity, n. (%)	10 (23)	17 (37)	0.14
Family history of thyroid cancer, n. (%)	14 (14)	9 (12)	0.66
Thyroid function, n. (%)			0.24
Euthyroidism	66 (87)	48 (92)	
Hypothyroidism	6 (8)	4 (8)	
Multinodular goiter, n. (%)	70 (71)	54 (71)	0.96
**Ultrasonographic features**			
Echogenicity, n. (%)			< 0.001
Solid isoechoic	39 (39)	13 (17)	
Partially cystic nodule	31 (31)	14 (18)	
Solid hypoechoic	29 (29)	49 (64)	
Margins, n. (%)			< 0.001
Regular	97 (98)	57 (75)	
Irregular	2 (2)	19 (25)	
Nodule size, mm (mean ± SD)	23.6 ± 12.1	19.9 ± 13.0	0.052
Taller than wide shape, n. (%)	2 (2)	7 (9)	0.033
Microcalcifications	1 (1)	19 (25)	< 0.001
Macrocalcifications	4 (4)	10 (13)	0.028
**Cytological features**			
Colloid, n. (%)	45 (45)	24 (32)	0.063
Macrophages, n. (%)	39 (39)	25 (33)	0.38
Oncocytic cells, n. (%)	46 (46)	31 (41)	0.45
Inflammation, n. (%)	69 (70)	46 (61)	0.21
Fibrosis, n. (%)	43 (43)	26 (34)	0.22
Chromatin clearing, n. (%)	90 (91)	75 (99)	0.028
Fusiform nuclei, n. (%)	79 (80)	58 (76)	0.58
Nuclear grooves, n. (%)	16 (16)	36 (47)	< 0.001
Nuclear inclusions, n. (%)	2 (2)	13 (17)	< 0.001
Microfollicular structures, n. (%)	88 (89)	58 (76)	0.027
Trabecular structures, n. (%)	71 (72)	44 (58)	0.056

#### Identification of ultrasonographic and cytological factors associated with malignancy in patients with TIR 3B nodules

Table 2 summarizes the ultrasonographic factors associated with malignancy in patients with TIR 3B nodules. Multivariable analysis identified solid hypoechoic nodules (OR: 4.26, 95% CI: 1.65–11.00; *p* = 0.003), irregular margins (OR: 8.35, 95% CI: 1.68–41.49; *p* = 0.009), microcalcifications (OR: 25.49, 95% CI: 3.07–211.93; *p* = 0.003), and macrocalcifications (OR: 5.72, 95% CI: 1.53–21.43; *p* = 0.010) as features associated with malignancy. Table 3 summarizes the cytological factors associated with malignancy in patients with TIR 3B nodules. Multivariable analysis identified nuclear grooves (OR: 3.72, 95% CI: 1.70–8.13; *p* = 0.001) and nuclear inclusions (OR: 9.06, 95% CI: 1.76–46.49; *p* = 0.008) as features associated with malignancy. A multivariable analysis incorporating both significant ultrasonographic and cytological features was conducted (Table 4). This analysis confirmed that solid hypoechoic nodules (OR: 3.39, 95% CI: 1.56–7.38; *p* = 0.002), irregular margins (OR: 8.93, 95% CI: 1.81–44.98; *p* = 0.007), nuclear grooves (OR: 4.11, 95% CI: 1.79–9.43; *p* = 0.001), microcalcifications (OR: 15.12, 95% CI: 1.86–123.05; *p* = 0.011), and macrocalcifications (OR: 4.83, 95% CI: 1.26–18.57; *p* = 0.022) were strongly associated with malignant TIR 3B nodules.

**Table 2:** Logistic regression analysis to evaluate ultrasonographic factors associated with malignancy in patients with TIR 3B nodules

	Multivariable analysis		
	Odds ratio	95% CI	*P* value
Solid isoechoic nodule	1.58	0.58–4.33	0.37
Solid hypoechoic nodule	4.26	1.65-11.00	0.003
Irregular margins	8.35	1.68–41.49	0.009
Nodule size	1.01	0.98–1.04	0.550
Taller than wide shape	1.09	0.16–7.52	0.926
Microcalcifications	25.49	3.07-211.93	0.003
Macrocalcifications	5.72	1.53–21.43	0.010

**Table 3:** Logistic regression analysis to evaluate cytological factors associated with malignancy in patients with TIR 3B nodules

	Multivariable analysis		
	Odds ratio	95% CI	*P* value
Colloid	0.45	0.22–0.93	0.032
Chromatin clearing	3.66	0.43–30.81	0.233
Nuclear grooves	3.72	1.70–8.13	0.001
Nuclear inclusions	9.06	1.76–46.49	0.008
Microfollicular structures	0.61	0.23–1.60	0.315
Trabecular structures	0.49	0.23–1.04	0.062

**Table 4:** Logistic regression analysis to evaluate cytological and ultrasonographic factors associated with malignancy in patients with TIR 3B nodules

	Multivariable analysis		
	Odds ratio	95% CI	*P* value
Solid hypoechoic nodule	3.39	1.56–7.38	0.002
Nuclear grooves	4.11	1.79–9.43	0.001
Irregular margins	8.93	1.81–44.98	0.007
Microcalcifications	15.12	1.86-123.05	0.011
Macrocalcifications	4.83	1.26–18.57	0.022

#### Predictive model for malignancy risk in TIR 3B thyroid nodules

**Fig. 2:**
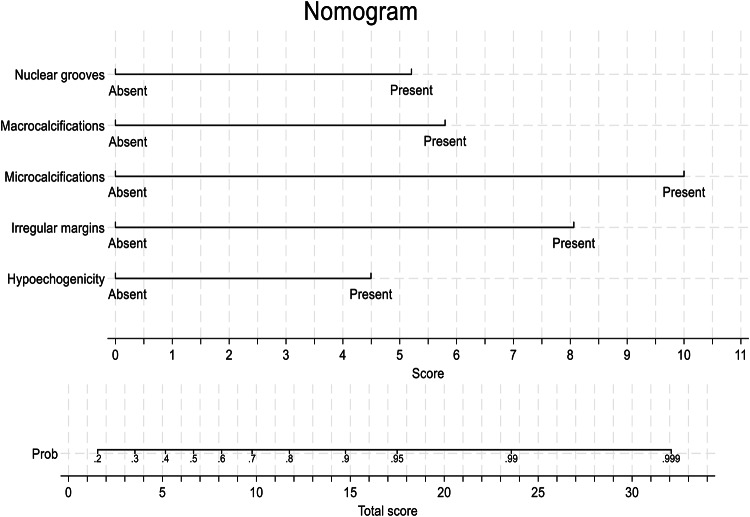
Nomogram for predicting malignancy risk based on independent factors. Each factor is assigned a score depending on its presence. Nuclear grooves: 5.2 points; Macrocalcifications: 5.8 points; Microcalcifications: 10.0 points; Irregular margins: 8.1 points; Hypoechogenicity: 4.5 points. The total score corresponds to the probability of malignancy shown at the bottom scale

According to the final results of the multivariable analysis, variables such as solid hypoechoic nodules, irregular margins, nuclear grooves, and micro- and macrocalcifications were incorporated to develop a nomogram model (Fig. 2). This model aims to facilitate the prediction of malignant TIR 3B nodules. The nomogram illustrates factors independently associated with malignancy by assigning a specific score to each factor based on its presence or absence. “Absent” corresponds to a score of zero, while “Present” adds a defined value. Specifically, nuclear grooves corresponded to 5.2 points, macrocalcifications to 5.8 points, microcalcifications to 10.0 points, irregular margins to 8.1 points, and hypoechogenicity to 4.5 points. The total score is calculated by summing the contributions of all factors. The total score is calculated by summing the contributions of all factors. Once determined, the total score is plotted on the “Total Score” axis at the bottom of the chart. This value is then mapped to the Probability (“Prob”) scale, which estimates the likelihood of malignancy. The relationship between the total score and probability is shown continuously, with higher scores indicating a greater likelihood of malignancy. The area under the curve (AUC) of the Receiver Operating Characteristic (ROC) analysis was 0.790, reflecting good overall diagnostic performance, with a sensitivity of 63% and a specificity of 94%. To assess the predictive performance of the new algorithm, a ROC curve analysis was conducted using the histological outcome as the reference standard. This analysis evaluates the diagnostic accuracy of the total score in distinguishing between benign and malignant cases. An optimal cut-off score of 8 was identified: scores above this threshold are more likely to indicate malignancy. Scores below 8 are associated with a lower risk of malignancy, as most nodules (76.9%) are benign, while 23.1% are malignant. Conversely, scores of 8 or higher indicate a higher malignancy risk, with most nodules (88.9%) being malignant and only 11.1% classified as benign.

## Discussion

Our study highlights the importance of integrating clinical, ultrasonographic, and cytological characteristics to enhance the prediction of malignancy in TIR 3B thyroid nodules and reduce unnecessary surgical interventions. The development of a nomogram model based on multivariable analysis provides clinicians with an evidence-based tool for improved risk stratification. Introduced in 2014 by the ICCRTC, the Italian cytological classification of thyroid nodules has proven effective in distinguishing indeterminate lesions with a low risk of malignancy from those with a high risk: follow-up is recommended for the newly categorized low-risk indeterminate lesion, while surgery is advised for the high-risk indeterminate lesion [21, 28, 29]. However, despite its strong performance in identifying malignant nodules, nearly half of patients with a TIR 3B cytological diagnosis undergo unnecessary surgery for lesions that ultimately turn out to be benign. Our results highlighted several key findings regarding malignancy risk and identified specific ultrasonographic and cytological features associated with malignant outcomes to help determine which patients should be candidates for surgery. Based on these significant ultrasonographic and cytological features, we developed a nomogram to estimate malignancy risk in TIR 3B nodules. This predictive model demonstrated good diagnostic performance, with an AUC of 0.790. The identified cut-off score of 8 effectively stratified nodules into high- and low-risk groups. Nodules with scores ≥ 8 were predominantly malignant (88.9%), whereas those with scores below 8 were mostly benign (76.9%). The high specificity (94%) observed in our model suggests that this scoring system could be a valuable tool for guiding surgical decisions. By accurately identifying patients harboring malignant thyroid nodules, the model facilitates timely surgical intervention for those who would benefit most, while simultaneously minimizing the number of false positives. Other authors have also attempted to develop diagnostic scoring systems to predict malignancy risk in indeterminate thyroid nodules. Ulisse et al. proposed stratifying the malignancy risk of indeterminate lesions by combining ultrasound features with the SIAPEC 2014 classification, identifying thyroid nodules with low, intermediate, and high risk [30]. Similarly, Ianni et al. developed and validated a scoring system that integrates US and clinical risk factors to identify thyroid nodules with low, intermediate and high risk of malignancy [31]. Similarly to our approach, a recent study by Sparano et al. evaluated clinical, ultrasonographic, and cytological variables, developing a scoring for predict the risk of malignancy in TIR 3B nodules [32]. Their algorithm predicted adverse outcomes with good accuracy (AUC = 0.748), suggesting that some TIR 3B nodules, with a very low score, can be clinically followed up without the immediate need for surgical intervention.

**Fig. 3:**
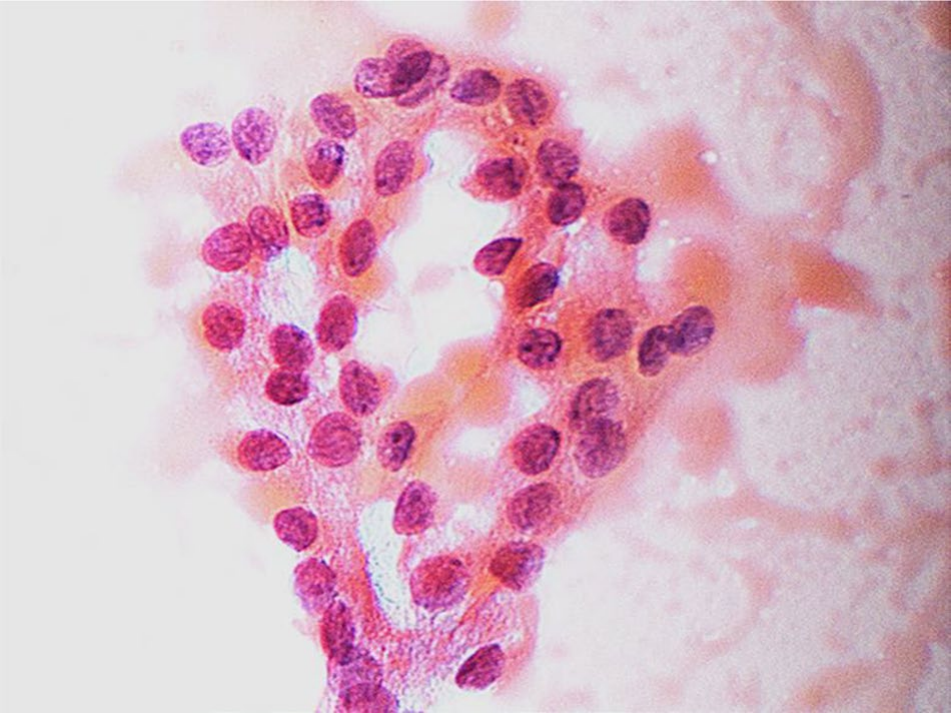
The image shows thyroid follicular cells with irregular nuclei and prominent nuclear grooves in a cytological slide of a nodule classified as TIR 3B. Histological examination subsequently confirmed the diagnosis of papillary thyroid carcinoma

The high prevalence of malignancy observed in TIR 3B nodules in this study (43.4%) is consistent with the malignancy rate reported in recent studies [32, 33]. These findings highlight the need for a more tailored and evidence-based approach to the management of TIR 3B nodules. In our study, all patients with TIR 3B nodules were referred to surgery, according to current guidelines [2, 21]. We found that younger age at diagnosis was significantly associated with malignancy. This is consistent with previous study which have found that patients with benign nodules are older than those with malignant ones [34]. Specifically, in the study of Cozzolino et al., the authors found that age < 55 years was an independent risk factor for thyroid cancer [23]. Among the ultrasonographic features, we found that hypoechogenicity, irregular margins, micro- and macrocalcifications were independently associated with malignancy. These findings are consistent with the American Thyroid Association (ATA) and European Thyroid Association (ETA) guidelines, which identify these features as high-risk indicators for thyroid malignancy [2, 9]. In addition, in the study of Guarnotta et al. the authors identified intranodular and perivascular flows and nodule size ≤ 11 mm were strongly associated with TIR 3B nodules, highlighting the importance of the vascular nodule flow [35]. Several studies have focused attention on thyroid macrocalcifications with controversial results. Although some studies claim that macrocalcification associated with thyroid nodule is not a reliable criterion for malignancy, other reports identified a higher risk of thyroid cancer [36–39]. It should be noted that the predictive value of ultrasonographic features, although well established and validated, may vary depending on the characteristics of the analyzed cohort and the cytological context. In our series, which included only TIR 3B nodules, the relative weight of each ultrasound feature within the nomogram reflects the specific distribution of cytological atypia and other coexisting risk factors. Therefore, the contribution of each parameter should be interpreted in light of the population studied. Among cytological features, we found that colloid was associated with benign nodules while nuclear grooves and inclusions the opposite. Nuclear grooves (Fig. 3), often referred as “coffee-bean nuclei” due to resemblance to coffee beans, are longitudinal nuclear membrane invaginations or folds along the long axis of the nucleus [40]. Consistent with findings reported in the literature, our study identified nuclear grooves as an independent predictor of malignancy [41]. However, although nuclear grooves are commonly associated with PTC, they can also be found in various other thyroid conditions such as diffuse hyperplasia, Hashimoto’s thyroiditis and non-papillary thyroid neoplasms; indeed, in the study of Cozzolino et al. the presence of nuclear grooves was similar between thyroid cancer e benign nodules [23, 40, 41]. Contrary to previous features, according to other studies, the presence of colloid is a clear sign of benign thyroid nodule [32, 42]. This study has several limitations. First, its retrospective nature may introduce selection bias. Second, the relatively small sample size and the monocentric setting may limit the generalizability of our findings. Larger, prospective studies are needed to validate our predictive model and confirm its utility in clinical practice. Moreover, peri- and intralesional vascular flows were not evaluated in this study, which, as seen above, are useful tools for nodular characterization. Finally, the sensitivity of the nomogram does not allow for the accurate exclusion of benign TIR3B nodules, thereby limiting its ability to correctly identify true negatives. As a result, while the nomogram effectively detects malignant lesions, it is less reliable in ruling out benign ones. In conclusion, the nomogram developed by combination of clinical, ultrasonographic and cytological features can be a simple and useful tool for predicting the risk of malignancy of nodules with indeterminate cytology. This model could be a valuable clinical tool for guiding more tailored management decisions and improving outcomes for patients with indeterminate high-risk thyroid nodules.

## Data Availability

The data sets used and/or analyzed during the current study are available from the corresponding author on reasonable request.
